# Targeting circRNA-MAP4K2 for the treatment of diabetes-induced retinal vascular dysfunction

**DOI:** 10.18632/aging.204215

**Published:** 2022-08-11

**Authors:** Cong Ma, Ze-Hui Shi, Xiao-Yan Han, Chang Liu, Biao Yan, Jian-Ling Du

**Affiliations:** 1 Department of Endocrinology, The First Affiliated Hospital of Dalian Medical University, Dalian, Liaoning Province, China; 2 Department of Ophthalmology, The First Affiliated Hospital of Dalian Medical University, Dalian, Liaoning Province, China; 3 Eye Institute, Eye and ENT Hospital, Shanghai Medical College, Fudan University, Shanghai, China

**Keywords:** diabetic retinopathy, retinal vascular complication, circular RNA, endothelial cell, angiogenic effect

## Abstract

Diabetic retinopathy (DR) is an important ocular vascular disease in working-age adults. However, the molecular mechanism underlying retinal vascular dysfunction is still not fully understood in DR. Circular RNAs have been recognized as the crucial regulators in many biological processes and human diseases. Herein, we determined the role of circular RNA-MAP4K2 (cMAP4K2) in diabetes-induced retinal vascular dysfunction. The results showed that high glucose treatment led to increased levels of cMAP4K2 expression *in vitro* and *in vivo*. Silencing of cMAP4K2 could reduce endothelial cell viability, proliferation, migration, and tube formation *in vitro* and alleviate retinal vascular dysfunction *in vivo* as shown by decreased vascular leakage and inflammation. By contrast, cMAP4K2 overexpression had an opposite effect on retinal vascular dysfunction. Mechanistically, cMAP4K2 acted as miR-377 sponge to affect the biological activity of miR-377, which led to increased expression of vascular endothelial growth factor A (VEGFA). Clinically, cMAP4K2 expression was significantly up-regulated in the clinical sample of DR patients. Collectively, cMAP4K2 is shown as a potential target for the diagnosis and treatment of diabetic retinopathy.

## INTRODUCTION

Diabetic retinopathy (DR) is an important ocular vascular disease in working-age adults [1]. Vision impairment is primarily caused by retinal vascular complications, including hyperpermeability, hypoperfusion, and angiogenesis. These pathological changes are tightly associated with the anatomical and functional changes of retinal vessels [2, 3]. Nowadays, laser photocoagulation, vitreous surgery, and anti-VEGF drugs have been used for DR treatment [4]. However, these treatment options only act at the advanced stage of DR, have short-term efficacy, and may cause several side effects, such as retinal detachment, intraocular infection, and raised IOP (intraocular pressure) [5, 6]. Thus, further studies are still required to clarify the underlying mechanism of DR.

Eukaryotic circular RNAs (circRNAs) are produced from the precursor mRNAs via back-splicing events [7]. They have covalently closed loops and control gene expression by affecting mRNA transcription, protein activity, and miRNA function [8]. circRNAs often exhibit differential expression pattern across distinct species, developmental stages, and pathologies [9]. Recently, circRNAs have been identified as the biomarkers in some vascular diseases and neurodegenerative diseases, such as atherosclerosis, coronary artery disease, stroke, and Alzheimer’s disease [10–13]. Considering the crucial role of circRNAs in the physiological and pathological processes, it is not surprising that circRNAs are the potential targets for DR treatment.

In this study, we clarified the role of cMAP4K2 in retinal vascular dysfunction in DR. We revealed that the expression of cMAP4K2 was obviously induced in retinal endothelial cells and retinal vessels upon high glucose stress. Inhibition of cMAP4K2 could retard the development of diabetes-induced retinal vascular dysfunction. cMAP4K2 may serve as a potential target for DR treatment.

## MATERIALS AND METHODS

### Diabetic mice model

C57BL/6 mice (6–8 weeks old, weighing 20–25 g, male) were fasted for about 6 h, and then received an intraperitoneal (i.p.) injection of 75 mg/kg streptozotocin (STZ, Sigma-Aldrich, USA) or vehicle (0.1 M citrate buffer, pH 4.5) for building diabetic model. FBG (fasting blood glucose) level was detected using the blood collected from the tail vein. FBG ≥ 16.7 mmol/L was considered hyperglycemic [14].

### Isolation of retinal vessels

Retinas were incubated with 1 mL of deionized water and gently rotated for 60 min at room temperature. Then, 200 U DNase I Type II (Sigma-Aldrich, USA) was used to dissociate the lysed cell debris from retinal vessels for 10 min at 37°C. Retinal vessels were then incubated with the Dynabeads (Invitrogen, USA) conjugated with mouse anti-PECAM-1 antibody (BD Bioscience, USA) for 30 min at 4°C, followed by incubating with the sheep anti-mouse IgG-conjugated magnetic beads (Dynabead; Invitrogen, USA). Retinal vessels were obtained via magnetic separation and lysed for RNA extraction [15].

### HRVEC culture and transfection

Human retinal vascular endothelial cells (HRVECs) were obtained from Cell Systems Corporation and maintained in endothelial cell culture medium with 10% fetal bovine serum (FBS) with 5% CO_2_ at 37°C. Cell passage was performed when the coverage reached about 90% of culture flask. The cells at passages 5–8 were used. HRVECs were seeded onto the 24-well plates 24 h before transfection. siRNAs were transiently transfected into HRVECs using Lipofectamine 3000 (Thermo Fisher Scientific, USA). The target sequence for designing cMAP4K2 siRNA was 5′-GGGATGCTCAGGTGCCAGTGT-3′.

### RNA extraction and qRT-PCR assay

Total RNAs were isolated from retinal vessels, HRVECs, and clinical ocular samples by the TRIzol reagent (Invitrogen, USA). The total RNAs were then reversely transcribed into cDNAs using the SuperScript First Strand cDNA System (Invitrogen, USA). SYBR Premix Ex Taq II kit (Takara, Japan) was used for qPCR assays, which were performed on a PikoReal Real-Time PCR System (Thermo Fisher Scientific, USA). Relative gene expression was detected using the 2^−ΔΔCt^ method.

### MTT assay

The viability of HRVECs were detected by MTT assays. HRVECs (5 × 10^3^ cells/well) were seeded into 96-well plates for 12 h. Then, 0.5% MTT solution (Sigma-Aldrich, USA) were added and incubated with HRVECs for 3 h. After removing cell medium, DMSO solution (Sigma-Aldrich, USA) was added into each well. Finally, the absorbance at 495 nm was recorded using a microplate reader (Molecular Devices, USA) after the formazan was totally dissolved [11].

### CCK-8 assay

Cell viability was also detected using the Cell Counting Kit-8 kit (CCK-8; Solarbio, China). About 5 × 10^3^ cells/cell were seeded into 96-well plates. After the required treatment, 100 μL of 10% CCK-8 reagent was added to each well. HRVECs were incubated with CCK-8 reagent for 1.5 h at 37°C. The absorbance was determined at 450 nm wavelength by a microplate reader (Molecular Devices, USA) [16].

### Cell proliferation assay

The proliferation ability was determined using Ki67 immunofluorescence staining [11]. HRVECs were fixed in 4% paraformaldehyde (PFA, Biosharp, China) for 20 min and blocked with 5% BSA for 0.5 h. Ki67 antibody (Abcam, USA) was then incubated with HRVECs at 4°C overnight, following by incubating with Cy3-conjugated antibody (Life Technologies, USA) at room temperature for 2 h. Cell nuclei were visualized by 4′, 6-diamidino-2-phenylindole (DAPI, C1002, Beyotime, China) staining. Finally, the results of Ki67 staining were observed under a microscope.

### Cell migration assay

The migration ability was detected using the transwell chamber with 8 μm pore filters. Briefly, HRVECs were suspended in DMEM medium and about 1 × 10^5^ cells were cultured into the upper chamber. Meanwhile, the complete medium was added into the lower chamber. After the incubation, HRVECs were fixed in 4% PFA for 0.5 h. These cells on the upper surface were removed using the cotton swabs. The migrated cells in the lower chamber were stained with 0.5% crystal violet (Sigma-Aldrich, USA) and counted under a microscope [17].

### Tube formation assay

The pre-cooled 24-well plate was pre-coated with growth factor reduced Matrigel (BD Biosciences, USA) for 30 min at 37°C. After the required treatment, HRVECs (1 × 10^5^ cells/well) were seeded onto the Matrigel and incubated at 37°C/5% CO_2_/95% humidity for 6 h. Finally, the cumulative tube lengths were measured and quantified by image J software [18].

### RNA pull-down assay

RNA pull-down assay was conducted to investigate RNA-RNA interaction in HRVECs. In brief, 3′-end biotinylated miR-377 or 3′-end biotinylated miR-21 (RiboBio, China) were transfected into HRVECs for 8 h. Then, streptavidin-coated magnetic beads (Life Technologies, USA) were added by incubating with HRVEC lysates to obtain biotin-coupled RNA complex. Finally, the amount of cMAP4K2 or cSPECC1 in the bound fractions were examined by qRT-PCRs.

### Luciferase reporter assay

The entire cDNA sequence of cMAP4K2, VEGFA-3′-UTR or the mutant without miR-377-binding regions were cloned into the luciferase vector to generate cMAP4K2-WT, VEGFA-WT, and VEGFA-Mut vector. Then, these plasmids and miR-377 mimic were co-transfected into HRVECs, respectively. At 24 h after transfection, HRVECs were lysed and the dual-luciferase reporter kit (Promega, USA) was used to detect luciferase activity.

### RIP assay

Magna RIP RNA-binding protein immunoprecipitation kit (Millipore, USA) was used for RIP assay to detect RNA-protein interaction. Briefly, HRVECs were lysed in the RIP lysis buffer and then divided into two fractions. These fractions were then incubated with IgG-conjugated magnetic beads (Millipore, USA) or Ago2-conjugated magnetic beads (Abcam, USA) at 4°C overnight. Finally, the bound RNAs were purified and qRT-PCRs were used to detect the amount of cMAP4K2 and miR-377 in the bound RNAs [19].

### Detection of retinal vessel permeability

Evans blue assays were employed to examine retinal vessel permeability. Two hours after intraperitoneal injection of Evans blue dye (75 mg/kg), the mice were anesthetized using the mixture of ketamine (80 mg/kg) and xylazine (4 mg/kg). Then, PBS buffer was used for perfusion to remove Evans blue dye. Euthanasia was conducted by carbon dioxide and the eyeballs were enucleated. After fixing the eyeballs with 4% PFA for 1 h, the retinas were cut and flat-mounted [17].

### Enzyme-linked immunosorbent assay (ELISA)

ELISA kit (R&D Systems) was used to examine the expression of IL-2, IL-6, and TNF-α in retinal lysates by following the protocol for manufacturing. The optical density was detected at 450 nm wavelength and plotted based on the numerical values.

### Clinical samples collection

We collected vitreous samples from the patients with idiopathic macular hole (non-DR, *n* = 30 eyes) and PDR (*n* = 30 eyes) during pars plana vitrectomy. These samples were placed on an ice box, centrifuged to remove insoluble fractions, and stored at 80°C until further assays. We collected aqueous humor (AH) samples from the patients with diabetic retinopathy (*n* = 30 eyes) and the patients with cataract before surgery (*n* = 30 eyes). AH samples were centrifuged for 15 min and stored at −80°C until further assays.

### Data analysis

All data were analyzed by GraphPad prism version 6.0 and were shown as the mean ± standard error of the mean (SEM). For these normally distributed data, statistical significance was analyzed by Student *t* test, 1-way or 2-way ANOVA. For these non-normally distributed data, statistical significance was analyzed by Mann-Whitney *U* test or Kruskal-Wallis test.

## RESULTS

### cMAP4K2 expression is induced in retinal vessels and endothelial cells after high glucose exposure

**Figure 1:**
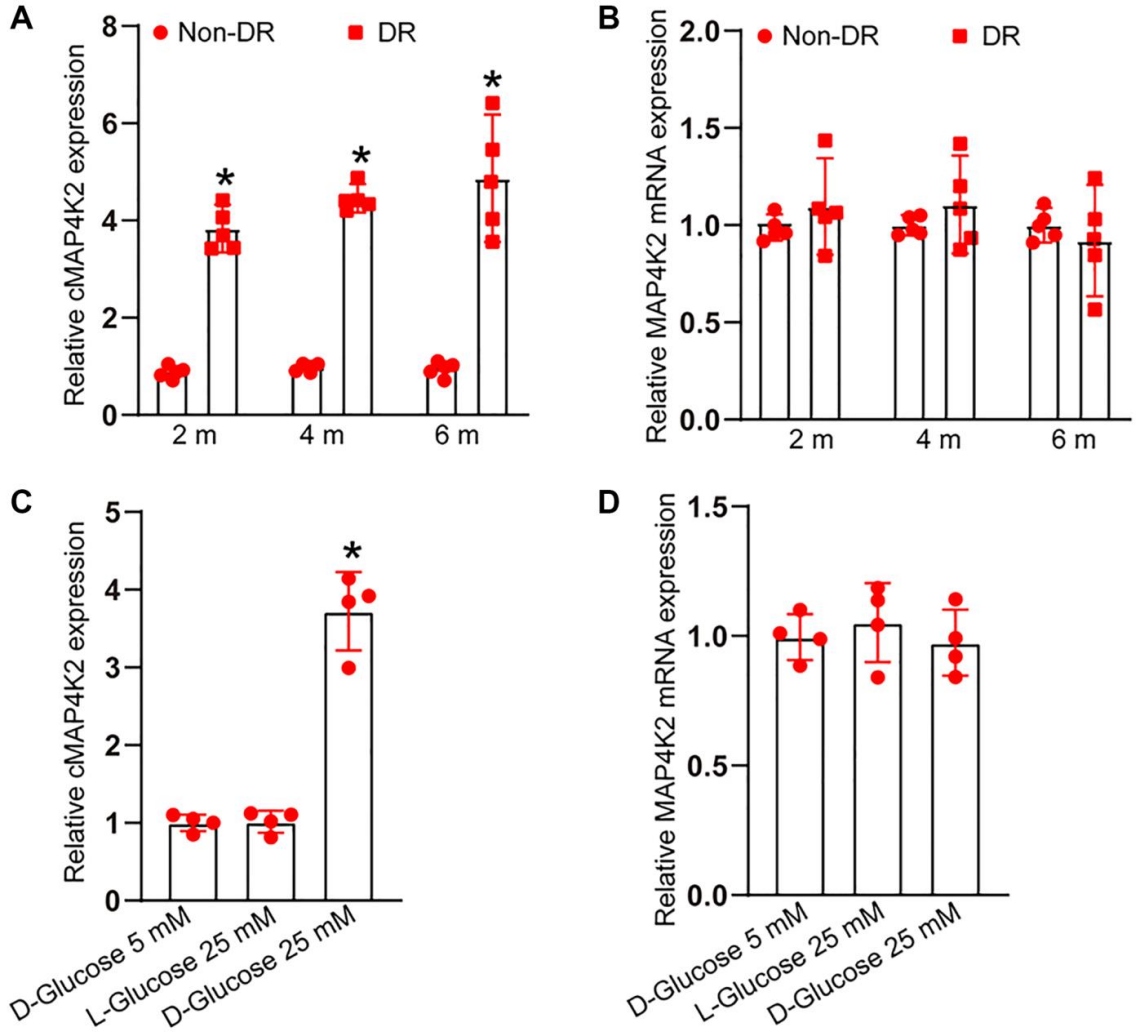
**cMAP4K2 expression is induced in retinal vessels and endothelial cells after high glucose exposure.** (**A** and **B**) qRT-PCRs were performed to detect the expression of cMAP4K2 and MAP4K2 mRNA in retinal vessels isolated from non-diabetic retinas (non-DR) and diabetic retinas (DR) at 2 month, 4 months, and 6 months (*n* = 5 animals per group, ^*^P < 0.05 vs. Non-DR group, Mann-Whitney *U* test). (**C** and **D**) qRT-PCRs were performed to detect the expression of cMAP4K2 and MAP4K2 mRNA in HRVECs cultured in the medium containing 5 mM glucose, L-glucose (25 mM), or D-glucose (25 mM) for 24 h (*n* = 4, ^*^P < 0.05 vs. 5 mM glucose group; 1-way ANOVA followed by Bonferroni’s post hoc comparison test).

To investigate the expression kinetics of cMAP4K2 during the progression of DR, STZ was administered to C57BL/6 mice to build the murine model of type 1 diabetes mellitus. Retinal vessels were extracted from non-diabetic retinas and diabetic retinas at 2-month, 4-month, and 6-month induction of diabetes. qRT-PCR assays indicated the expression of cMAP4K2 but not MAP4K2 mRNA was obviously induced in diabetic retinal vessels (Figure 1A and 1B). Given increased cMAP4K2 expression in diabetic retinal vessels, we next investigated whether hyperglycemia could trigger the induction of cMAP4K2 in retinal endothelial cells. HRVECs were exposed to normal culture media containing 5 mM glucose, D-glucose (25 mM), or L-glucose (25 mM). qRT-PCR assays indicated the expression of cMAP4K2 but not MAP4K2 mRNA was significantly induced in the hyperglycemia group (25 mM of D-glucose) compared with the normoglycemia group (5 mM glucose) or L-glucose group (Figure 1C and 1D). Thus, these results provided the rational to explore the role of cMAP4K2 in retinal vascular dysfunction in DR.

### cMAP4K2 regulates endothelial angiogenic effects *in vitro*

**Figure 2:**
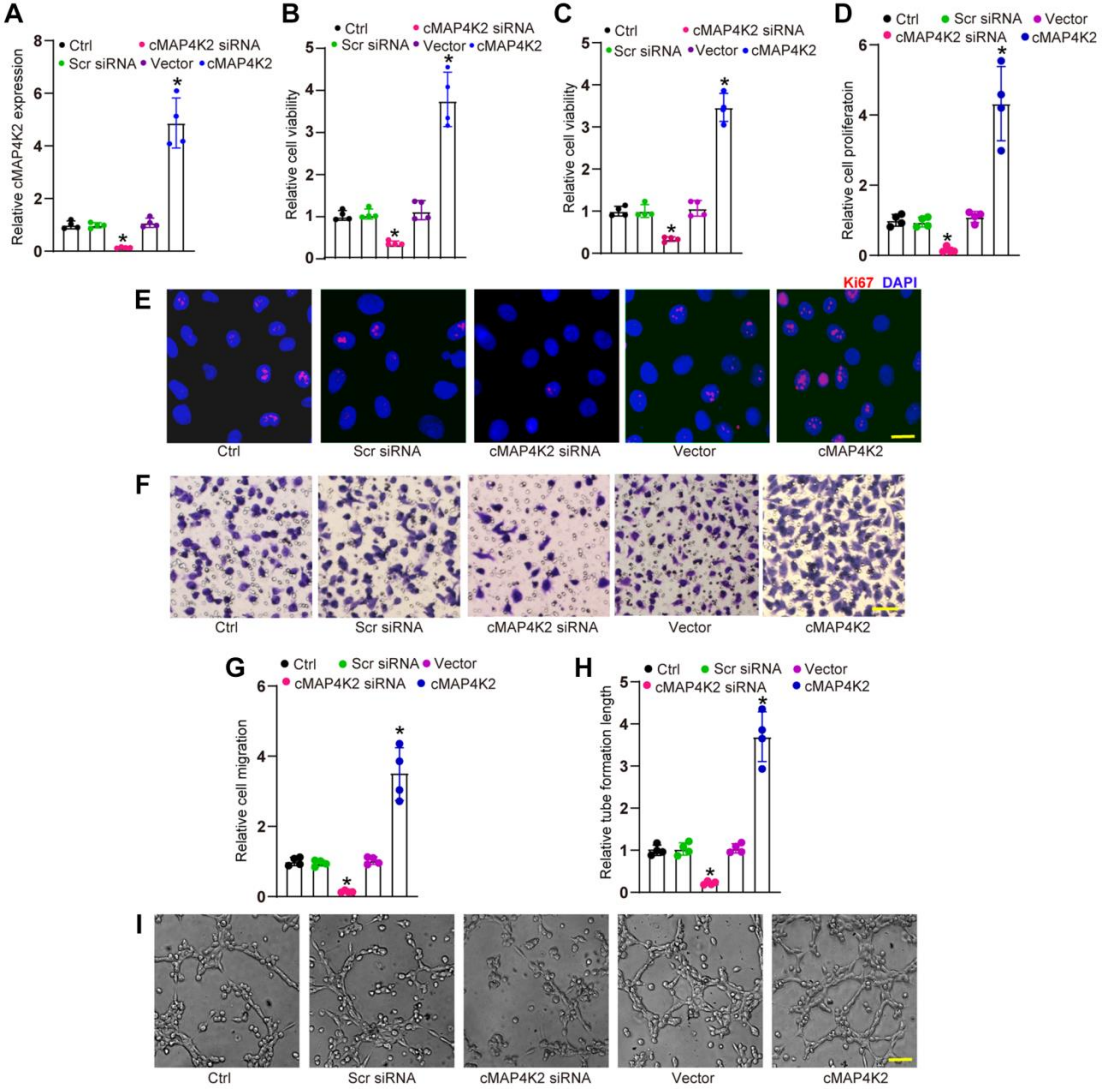
**cMAP4K2 regulates endothelial angiogenic effects *in vitro*.** (**A**) HRVECs were transfected with scramble (Scr) siRNA, cMAP4K2 siRNA, null vector, cMAP4K2 overexpressed vector, or left untreated (Ctrl) for 24 h. qRT-PCRs were performed to detect the expression levels of cMAP4K2 (*n* = 4, ^*^P < 0.05 vs. Ctrl group). (**B** and **C**) MTT and CCK-8 assays were performed to detect cell viability (*n* = 4, ^*^P < 0.05 vs. Ctrl group). (**D** and **E**) Ki67 staining assays were performed to detect cell proliferation (*n* = 4, ^*^P < 0.05 vs. Ctrl group). A representative image and quantification result were shown. Scale bar: 20 μm. (**F** and **G**) Transwell assay was performed to detect the migratory ability of HRVECs (*n* = 4, ^*^P < 0.05 vs. Ctrl group). A representative image and quantification result were shown. Scale bar: 50 μm. (**H** and **I**) The tube-like structures were observed at 6 h after seeding HRVECs on the Matrigel matrix. The cumulative tube lengths for each field were statistically analyzed (*n* = 4, ^*^P < 0.05 vs. Ctrl group). Scale bar: 100 μm. The significant difference was analyzed by one-way ANOVA followed by the Bonferroni post hoc test.

We then examined whether cMAP4K2 regulated endothelial angiogenic effects *in vitro*. Transfection of cMAP4K2 small interfering RNA (siRNA) led to reduced levels of cMAP4K2, while transfection of cMAP4K2 overexpression vector led to increased levels of cMAP4K2 in HRVECs (Figure 2A). MTT assays and CCK-8 assays revealed that transfection of cMAP4K2 siRNA decreased the viability of HRVECs (Figure 2B and 2C). Ki67 staining assays revealed that transfection of cMAP4K2 siRNA led to the reduction of HRVEC proliferation ability (Figure 2D and 2E). Transwell assays revealed that transfection of cMAP4K2 siRNA significantly decreased the migration ability of HRVECs (Figure 2F and 2G). Tube formation assays revealed that compared with control group, transfection of cMAP4K2 siRNA led to decreased cumulative tube length (Figure 2H and 2I). By contrast, cMAP4K2 overexpression led to the enhancement of cell viability, increased proliferative ability, accelerated migration ability, and enhanced tube formation ability (Figure 2B–2I). Thus, cMAP4K2 is shown as a critical regulator of endothelial angiogenic effects.

### cMAP4K2 regulates retinal vascular dysfunction in DR

**Figure 3:**
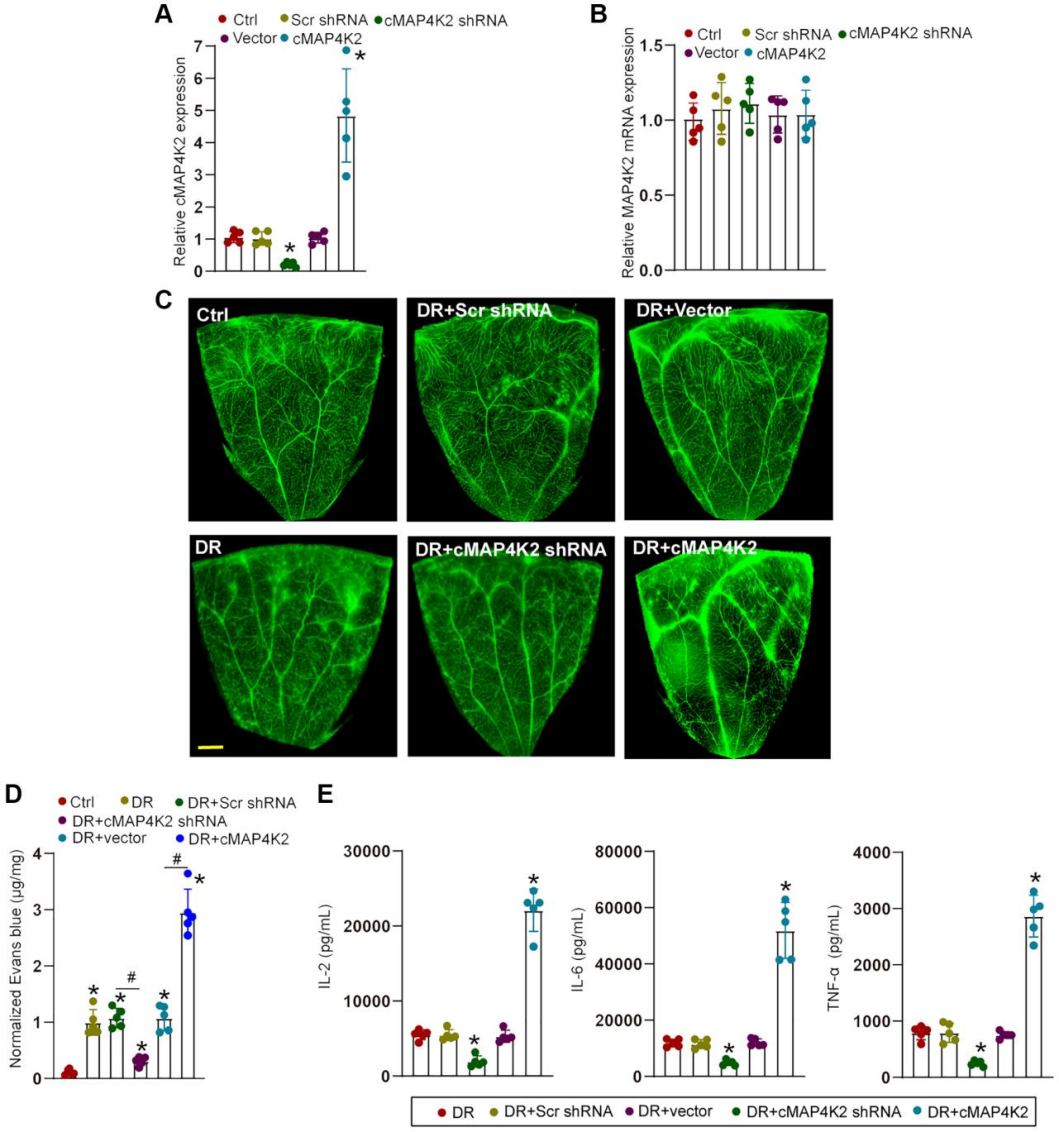
**cMAP4K2 regulates retinal vascular dysfunction in DR.** (**A** and **B**) C57BL/6 mice received an intraocular injection of scramble (Scr) shRNA, cMAP4K2 shRNA, null vector, cMAP4K2 overexpressed vector, or left untreated (Ctrl) for 2 weeks. qRT-PCR assays were performed to detect the expression levels of cMAP4K2 and MAP4K2 mRNA (*n* = 5, ^*^P < 0.05 vs. Ctrl group). (**C** and **D**) The mice were infused with Evans blue dye for 2 h. The fluorescence signal of flat-mounted retina was observed. A representative image and the quantification of Evans blue leakage was shown. Scale bar: 500 μm. (**E**) ELISA assays were performed to examine the amount of IL-2, IL-6, and TNF-α in retinal lysates among different groups (*n* = 5; ^*^P < 0.05 vs. DR group; ^#^*P* < 0.05 between the marked groups). The significant difference was analyzed by the Kruskal-Wallis test followed by the post hoc Bonferroni test.

We next determined the role of cMAP4K2 in retinal vascular dysfunction in DR. Intraocular injection of cMAP4K2 shRNA led to reduced levels of cMAP4K2 but did not alter expression levels of MAP4K2 mRNA in the retina. By contrast, intraocular injection of cMAP4K2 overexpression virus led to increased expression of cMAP4K2 in the retina, but also had no effects on MAP4K2 mRNA expression (Figure 3A and 3B). Evans blue assays demonstrated that injection of cMAP4K2 shRNA could alleviate diabetes-induced retinal vascular leakage in DR (Figure 3C and 3D). ELISA assays showed that injection of cMAP4K2 shRNA could reduce retinal inflammation responses as shown by decreased levels of interleukin (IL)-2, IL-6, and tumor necrosis factor-α (TNF-α) in DR (Figure 3E). By contrast, injection of cMAP4K2 overexpression vector could lead to increased retinal vascular leakage and aggravated retinal inflammation in DR (Figure 3C–3E).

### cMAP4K2 regulates endothelial cell function by acting as miR-377 sponge

**Figure 4:**
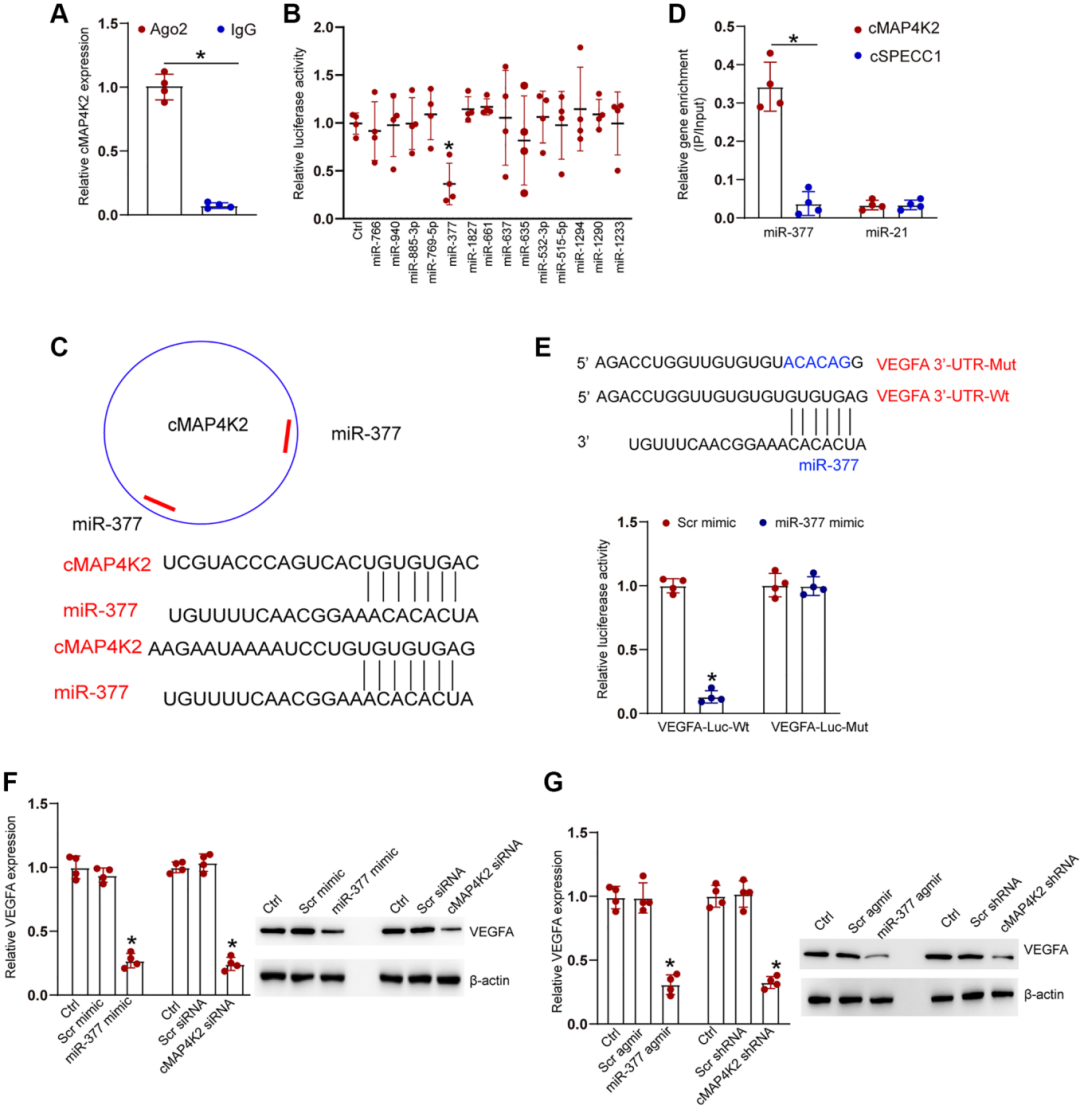
**cMAP4K2 regulates endothelial cell function by acting as the miRNA sponge.** (**A**) The cell fractions were isolated from HRVECs and immunoprecipitated using Ago2 or IgG antibody. The amount of cMAP4K2 in the immunoprecipitate was examined by qRT-PCRs (*n* = 4, ^*^P < 0.05). (**B**) HRVEC were transfected with pGL3-Basic (Ctrl) or LUC-cMAP4K2 with different miRNA mimic and pRL-TK vector (internal transfection control). Luciferase activity was detected at 36 h post-transfection using the Dual-Luciferase Reporter Assay kit (*n* = 4, ^*^P < 0.05). (**C**) The schematic figure showed the potential binding regions of miR-377 on cMAP4K2 transcript. (**D**) The biotinylated miR-21 or miR-377 were transfected into HRVECs. The amount of cMAP4K2 and cSPECC1 (negative control) in the input and bound fractions were examined by qRT-PCR assays following streptavidin capture. The relative immunoprecipitate (IP)/input ratios were plotted (*n* = 4). (**E**) LUC-VEGFA or LUC-VEGFA-mutant was co-transfected without or with miRNA mimic and pRL-TK vector. Luciferase activity was detected at 36 h after transfection (*n* = 4). MUT: mutated VEGFA 3’-UTR without miR-377 binding site. (**F**) HRVECs were cultured with D-glucose (25 mM) for 24 h and then transfected with scramble (Scr) mimic, miR-377 mimic, Scr siRNA, cMAP4K2 siNRA, or left untreated (Ctrl) for 24 h. qRT-PCRs and western blots were performed to detect the expression of VEGFA at mRNA levels and protein levels (*n* = 3). (**G**) Diabetic retinas received an injection of Src agomir, miR-377 agomir, Scr shRNA, cMAP4K2 shNRA, or left untreated (Ctrl) for 1-month. qRT-PCRs and western blots were performed to detect the expression of VEGFA at mRNA levels and protein levels (*n* = 3 animals per group). ^*^P < 0.05. The significant difference was analyzed by Student *t* test (**A**), one-way (**B**, **F**, and **G**), or two-way ANOVA (**D** and **E**) followed by the Bonferroni post hoc test.

We next studied the underlying mechanism of cMAP4K2 in endothelial cells. Previous studies have shown that circRNAs usually modulate gene expression by acting as miRNA sponges [11, 20]. Ago2 protein can bind miRNA complex to their target mRNAs. RNA immunoprecipitation (RIP) assays demonstrated that Ago2 antibody could specifically pull down cMAP4K2. By contrast, IgG could not specifically pull down cMAP4K2 transcript (Figure 4A). TargetScan algorithm was employed to identify the potential miRNAs which could interact with cMAP4K2 transcript. Luciferase activity screening showed that transfection of miR-377 mimics but no other miRNA mimics led to reduced luciferase activity of LUC-cMAP4K2 (Figure 4B). The potential binding regions of miR-377 on cMAP4K2 transcript were shown in Figure 4C. RNA pull-down assays demonstrated that cMAP4K2 was highly enriched in the miR-377-captured fraction compared with miR-21-captured fraction (negative control) (Figure 4D). VEGFA was predicted as the target gene of miR-377. The 3′-UTR of VEGFA was fused to luciferase reporter gene to obtain LUC-VEGFA vector. Luciferase activity assays demonstrated that overexpression of miR-377 significantly decreased the luciferase activity of LUC-VEGFA, whereas the mutation of miR-377-binding site within VEGFA prevented miR-377 mimic-mediated reduction of luciferase activity of LUC-VEGFA (Figure 4E).

We also detected the expression levels of VEGFA at the mRNA levels and protein levels following the expression manipulations of the upstream noncoding RNAs, such as cMAP4K2 and miR-377. qRT-PCR assays and western blots revealed that miR-377 overexpression or cMAP4K2 silencing caused a marked expression reduction of VEGFA at both transcript and protein levels in HRVECs and in diabetic retinas (Figure 4F and 4G).

### cMAP4K2-miR-377-VEGFA signaling axis regulates endothelial angiogenic effects

**Figure 5:**
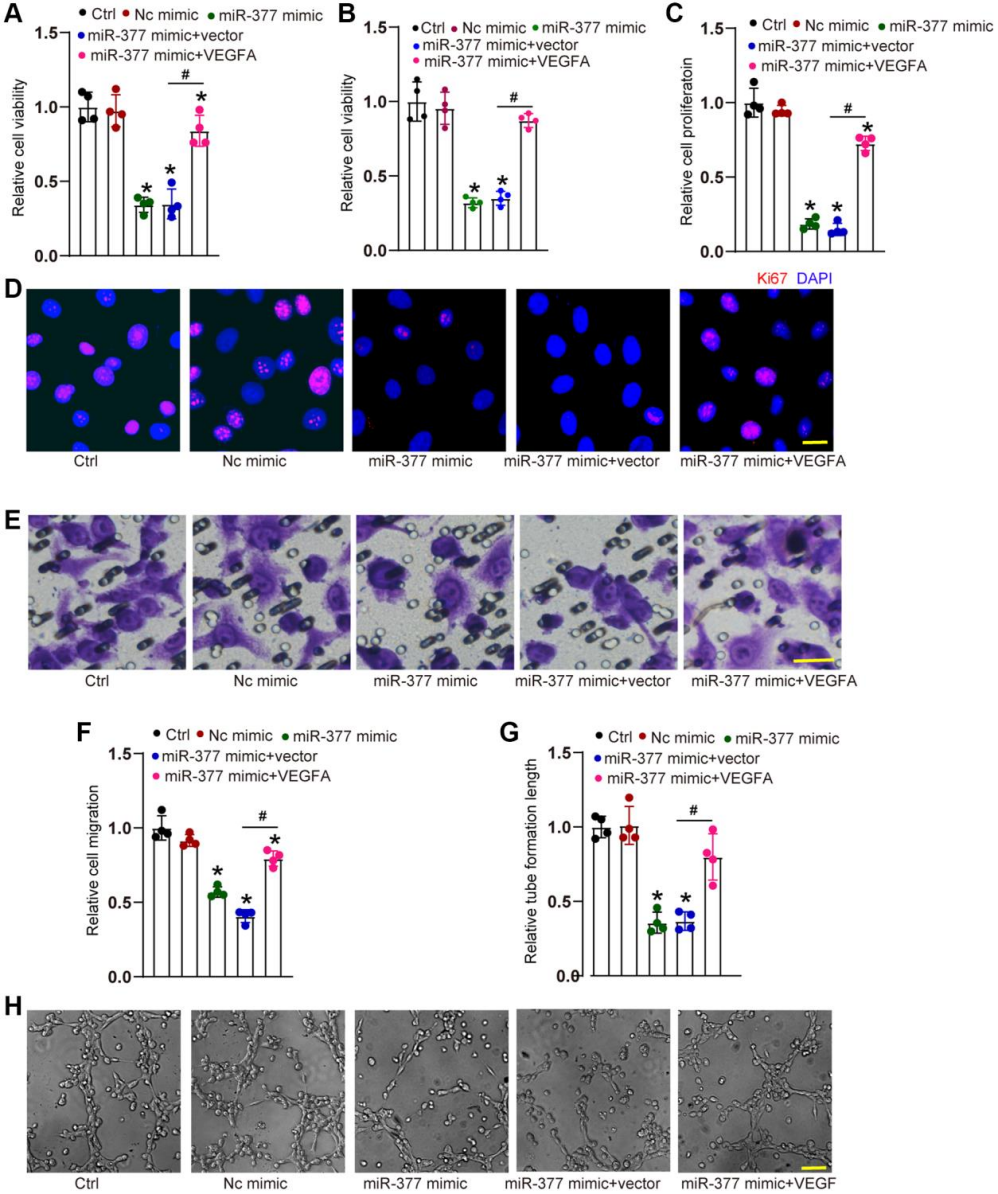
**cMAP4K2-miR-377-VEGFA signaling axis regulates endothelial angiogenic effects.** (**A**–**F**) HRVECs were treated as shown for 24 h. MTT assays and CCK-8 assays were performed to detect cell viability (**A** and **B**, *n* = 4). Ki67 staining assays were performed to detect cell proliferation. A representative image along with the quantification result were shown. Scale bar: 20 μm (**C** and **D**, *n* = 4). Transwell assay was performed to detect the migratory ability of HRVECs. A representative image and the quantification result were shown. Scale bar: 20 μm (**E** and **F**, *n* = 4). The tube-like structures were observed at 6 h after seeding HRVECs on the Matrigel matrix. The cumulative tube lengths for each field were analyzed. Scale bar: 100 μm (**G** and **H**, *n* = 4). ^*^P < 0.05 vs. Ctrl group. ^#^*P* < 0.05 miR-377 mimic + vector vs. miR-377 mimic + VEGFA. The significant difference was analyzed by one-way ANOVA followed by the Bonferroni post hoc test.

We also examined the role of miR-377 in endothelial angiogenic effects *in vitro*. Transfection of miR-377 mimic led to a marked reduction of cell viability (Figure 5A and 5B) as shown by MTT assays and CCK-8 assays, decreased proliferative ability (Figure 5C and 5D), reduced migration ability (Figure 5E and 5F), and reduced tube formation ability (Figure 5G and 5H). By contrast, overexpression of VEGFA could partially reverse the anti-angiogenic effects of miR-377 mimic on HRVEC as shown by increased cell viability, increased proliferative ability, accelerated cell migration, and enhanced tube formation ability (Figure 5A–5H). These results suggest that cMAP4K2-miR-377-VEGFA signaling axis regulates endothelial angiogenic function *in vitro*.

### Clinical significance of cMAP4K2-mediated signaling in DR

**Figure 6:**
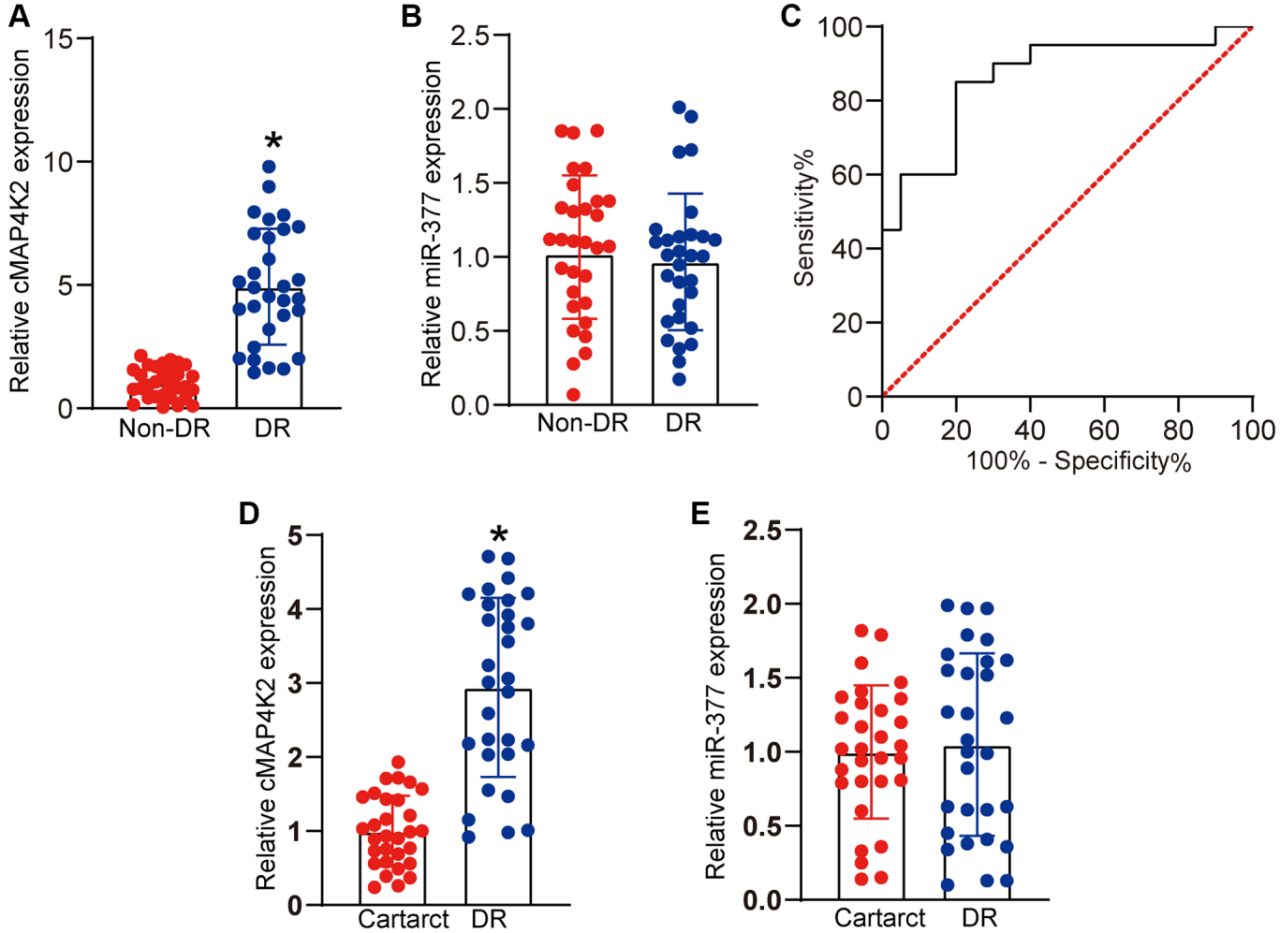
**Clinical significance of cMAP4K2-mediated signaling in DR.** (**A** and **B**) qRT-PCR assays were performed to detect the vitreous levels of cMAP4K2 and miR-377 in the patients with DR (*n* = 30 eyes) or non-diabetic patients with macular hole (non-DR, *n* = 30 eyes). (**C**) Receiver operating characteristic (ROC) curve analysis of cMAP4K2 and the corresponding area under curve (AUC) value in the discriminative power of cMAP4K2 for DR. ^*^P < 0.05 vs. non-DR group. The significant difference was analyzed by Student *t* test. (**D** and **E**) qRT-PCR assays were performed to detect the levels of cMAP4K2 and miR-377 in the aqueous humor of the patients with DR (*n* = 30 eyes) or cataract (*n* = 30 eyes). ^*^P < 0.05 vs. cataract group. The significant difference was evaluated by Student *t* test.

We thus studied the clinical significance of cMAP4K2-mediated signaling dysfunction in DR. Vitreous sample is the body fluid in ocular tissue, which can reflect the progression of ocular diseases [4]. qRT-PCR assays revealed that vitreous levels of cMAP4K2 were markedly up-regulated in DR patients (Figure 6A). By contrast, vitreous levels of miR-377 were not altered in DR patients (Figure 6B). We then determined the discriminative power of cMAP4K2 between non-DR controls and DR patients, the area under the ROC-AUC was calculate. The ROC-AUC of cMAP4K2 for differentiating DR patients from non-DR controls was 0.8625 (Figure 6C; 95% CI: 0.7452–0.9798). Aqueous humor (AH) was collected from the patients with DR or the patients with cataract before surgery. qRT-PCR assays revealed that AH levels of cMAP4K2 but not miR-377 were significantly up-regulated in DR patients (Figure 6D and 6E). Collectively, these results indicate that cMAP4K2 is a promising marker for the diagnosis of DR.

## DISCUSSION

DR is an important microvascular disorder caused by diabetes mellitus [21]. However, the precise mechanism underlying retinal vascular dysfunction remains largely unknown. In this study, we showed that cMAP4K2 expression was markedly induced in retinal vessels and retinal endothelial cells after high-glucose stress. Inhibition of cMAP4K2 could suppress endothelial angiogenic functions *in vitro* and alleviate retinal vascular complications in DR. The levels of cMAP4K2 were also up-regulated in the clinical samples of DR patients. Therefore, cMAP4K2 is shown as a potential regulator of DR pathogenesis and a promising biomarker for DR diagnosis.

Endothelial cells (ECs) can provide the interface between blood and vascular tissues. EC dysfunction contributes to the progression of diabetic vascular injury [22, 23]. Hyperglycemia leads to increased levels of cMAP4K2 in retinal ECs and retinal vascular tissues. Increased cMAP4K2 could alter the proliferation, migration, tube formation abilities of ECs. In the diseased condition, abnormal EC biology may affect retinal vascular remodelling [24, 25]. cMAP4K2 inhibition could reduce retinal vascular injuries as shown by decreased vascular leakage and reduced inflammation response. It is not surprising that increased cMAP4K2 level is a predisposing factor of diabetes-induced retinal vascular dysfunction.

circRNAs can provide a new post-transcriptional regulatory layer of gene expression by acting as miRNA sponge or protein sponge [26]. Previous studies have revealed that most of circular RNAs act as miRNA sponges. circMTO1 is shown as miR-9 sponge to inhibit hepatocellular carcinoma [27]. circZNF532 acts as miR-29a-3p sponge to regulate retinal pericyte degeneration and vascular degeneration [28]. circZNF609 acts as miR-615 sponge to affect retinal neurodegeneration [29]. In this study, RNA pull-down assay, luciferase activity assay, and qRT-PCR assay indicated that cMAP4K2 exerts its biological role via a similar mechanism. cMAP4K2 acts as the binding platform for Ago2 and miRNAs. It could act as miR-377 sponge to regulate the levels of VEGFA expression. Clinical efficacy of anti-VEGF drugs has widely applied for the treatment of ocular vascular disorders, such as diabetic macular edema and wet AMD [30]. Thus, cMAP4K2/miR-377/VEGFA signalling is tightly associated with retinal vascular dysfunction.

Retinal vascular dysfunction is highly complicated pathological process. Several pathways have shown to be involved in retinal vascular dysfunction, such as VEGF signalling, Wnt signalling, and Notch signalling. VEGF is considered as the primary driver of retinal vascular dysfunction. Moreover, anti-VEGF drugs are the major options for the treatment of pathological neovascularization in the eye [31, 32]. However, there are still 30%–40% patients which have no response to anti-VEGF treatment. In addition, anti-VEGF treatment may cause chorioretinal atrophy [33]. We reveal a novel mechanism underlying retinal vascular dysfunction which is different from the current VEGF signalling. Diabetes-induced cMAP4K2 up-regulation could act as an endogenous miRNA sponge for miR-377, inducing increased VEGFA expression. Increased VEGFA expression could lead to the activation of the downstream pathways, contributing to angiogenic effects, including abnormal proliferation, enhanced migration, and increased tube formation [34]. Thus, this finding provides a novel option for treating ocular vascular diseases.

Intraocular injection of anti-VEGF drugs, such as bevacizumab, pegaptanib, ranibizumab, and aflibercept have revolutionized the treatment efficiency for DR. Despite the promising results, intraocular injection of anti-VEGF drugs always causes several injection risks and adverse events. Repetitive and long-term injections may increase the chances of disease complications, such as endophthalmitis, retinal detachment, enhanced intraocular pressure, and hemorrhage [35]. In addition, intraocular injection of anti-VEGF drugs may enter into the body circulation and alter the levels of systemic VEGF, which could account for the systemic adverse events [36]. We identified a novel regulatory mechanism of VEGFA, which was mediated by cMAP4K2-miR-377-VEGFA regulatory axis. Based on this signalling axis, we could design the inhibitor of cMAP4K2 or miR-377 mimic, which are the small molecular drugs. Small molecular drugs are usually cheaper than antibody drugs. Moreover, they have low immunogenicity and are easily penetrated into intracellular targets and extracellular targets [37, 38]. Currently, small RNA-based therapeutics has become an emerging field for disease treatment. However, we should not ignore the off-target effects caused by the unintended interactions between small RNAs and cellular components, such as miRNA-like off-target effects resulting from partial sequence homology to the 3′-UTRs, potential immune response caused by delivery vehicle, or saturation effect of endogenous RNAi machinery [39, 40]. To overcome the off-target effects, we should consider improving small RNA delivery by designing novel gene carriers or conducting chemical modifications of small RNA molecules to reduce the potential off-target effects.

## CONCLUSION

This study reports that cMAP4K2 is shown as a crucial regulator of retinal vascular dysfunction in DR. cMAP4K2 can regulate endothelial angiogenic function and its intervention is helpful for the treatment of DR. Clinical evidence indicates that cMAP4K2 detection can differentiate DR patients from non-DR controls. Thus, cMAP4K2 is an emerging target for DR diagnosis and therapy.
